# Advances in measuring influenza burden of disease

**DOI:** 10.1111/irv.12533

**Published:** 2018-02-19

**Authors:** Vernon J. Lee, Zheng Jie Marc Ho, Ee Hui Goh, Harry Campbell, Cheryl Cohen, Vanessa Cozza, Julia Fitzner, Jorge Jara, Anand Krishnan, Joseph Bresee, the WHO Working Group on Influenza Burden of Disease

**Affiliations:** ^1^ Ministry of Health Singapore Singapore; ^2^ Saw Swee Hock School of Public Health National University of Singapore Singapore Singapore; ^3^ Centre for Global Health Research Usher Institute of Population Health Sciences University of Edinburgh Edinburgh UK; ^4^ Division of the National Laboratory Service Centre for Respiratory Diseases and Meningitis National Institute for Communicable Diseases Johannesburg South Africa; ^5^ Wits School of Public Health University of the Witwatersrand Johannesburg South Africa; ^6^ Global Influenza Programme World Health Organization Geneva Switzerland; ^7^ Center for Health Studies, Research Institute Universidad del Valle de Guatemala Guatemala City Guatemala; ^8^ Centre for Community Medicine All India Institute of Medical Sciences New Delhi India; ^9^ Influenza Division Centers for Disease Control and Prevention Atlanta GA USA

## 1 IMPORTANCE OF BURDEN OF DISEASE ESTIMATES

Influenza is a global public health threat, with seasonal and pandemic influenza resulting in substantial impact on health, the economy and society. The World Health Organization (WHO) has recently estimated that every year, 290 000 to 650 000 deaths are associated with respiratory diseases from seasonal influenza.^1^ This estimate takes into account findings from recent influenza respiratory mortality studies, including a study conducted by Iuliano et al.^2^ Many high‐income countries (HICs) that have invested in measuring the impact of influenza epidemics and the cost‐effectiveness of interventions against influenza have also spent substantial resources in preventing spread and mitigating health outcomes through vaccination, clinical management of severe cases and other public health measures. At the same time, many low‐ and middle‐income countries (LMICs), especially those in the tropics, are grappling with understanding the impact of influenza in their local setting, and to determine whether such interventions are cost‐effective vis‐à‐vis interventions for other diseases.^3^ Furthermore, LMICs are likely to have the highest burden of influenza in children, but these are also the countries with the least data available.^4^

## 2 EFFORTS TO EXPAND BURDEN OF DISEASE STUDIES

It is in this context that burden of disease studies are important to document the potential impact of influenza on various aspects of health. This includes the number of cases among higher‐risk populations, hospitalizations and deaths. Estimates of national disease burden during influenza seasons will raise awareness on the impact of seasonal influenza in the local setting. It will allow for cost‐effectiveness analyses to aid decision‐making on investments such as vaccination programmes, and can lead to an expansion of efforts to tackle the spread of influenza in regions where impact is highest. In countries that have conducted burden of disease and cost‐effectiveness studies, influenza vaccination programmes have likewise taken root.^5^, ^6^, ^7^, ^8^ Undertaking burden of disease studies for seasonal influenza also helps countries to develop surveillance and analytical capabilities for use during pandemics.

Previous influenza burden studies were performed mostly in HICs, whose results are not necessarily generalizable to LMICs because of differences in underlying determinants such as age structure, nutrition, prevalence of high‐risk conditions, uptake of preventive strategies and access to medical care. Influenza‐related research has been expanding in LMICs over the last few years, showing that influenza causes substantial clinical cases, hospitalizations and deaths across various geographical and social settings.^9^, ^10^, ^11^, ^12^, ^13^ Research has also shown that the outcomes from influenza infection may be more severe in LMICs compared to HICs,^14^, ^15^ and that LMICs contribute a disproportionate burden towards global influenza disease.^4^

However, influenza disease burden information is still sparse in regions such as Africa, Asia and South America where there are differences in way that healthcare services are organized, accessed and financed across countries, even within the same region. There is also a relative lack of data from the tropics, where influenza exhibits different seasonal patterns compared to temperate countries.^16^ In addition to national considerations, determining more accurate regional and global burden of disease estimates for influenza is necessary to provide perspective on the impact caused by influenza compared to other diseases, and to determine investments to develop better pharmaceuticals such as vaccines with broad immunogenicity and improved vaccine production methods. As the WHO global estimates are extrapolations from available national data, building accurate global estimates requires better data from representative areas from all regions of the world.

Since 2015, the WHO has been encouraging countries who have yet to embark on burden of disease studies to do so by publishing *A Manual for Estimating Disease Burden Associated with Seasonal Influenza* under the ambit of the Pandemic Influenza Preparedness (PIP) framework.^17^ It has also supported the conduct of such studies in LMICs in regions where little data existed through funding and provision of technical expertise.

This Special Edition of the Journal showcases the output of this ongoing work, which has led to a new series of seasonal influenza burden of disease studies.

## 3 NEW SERIES OF BURDEN STUDIES

**Figure 1:**
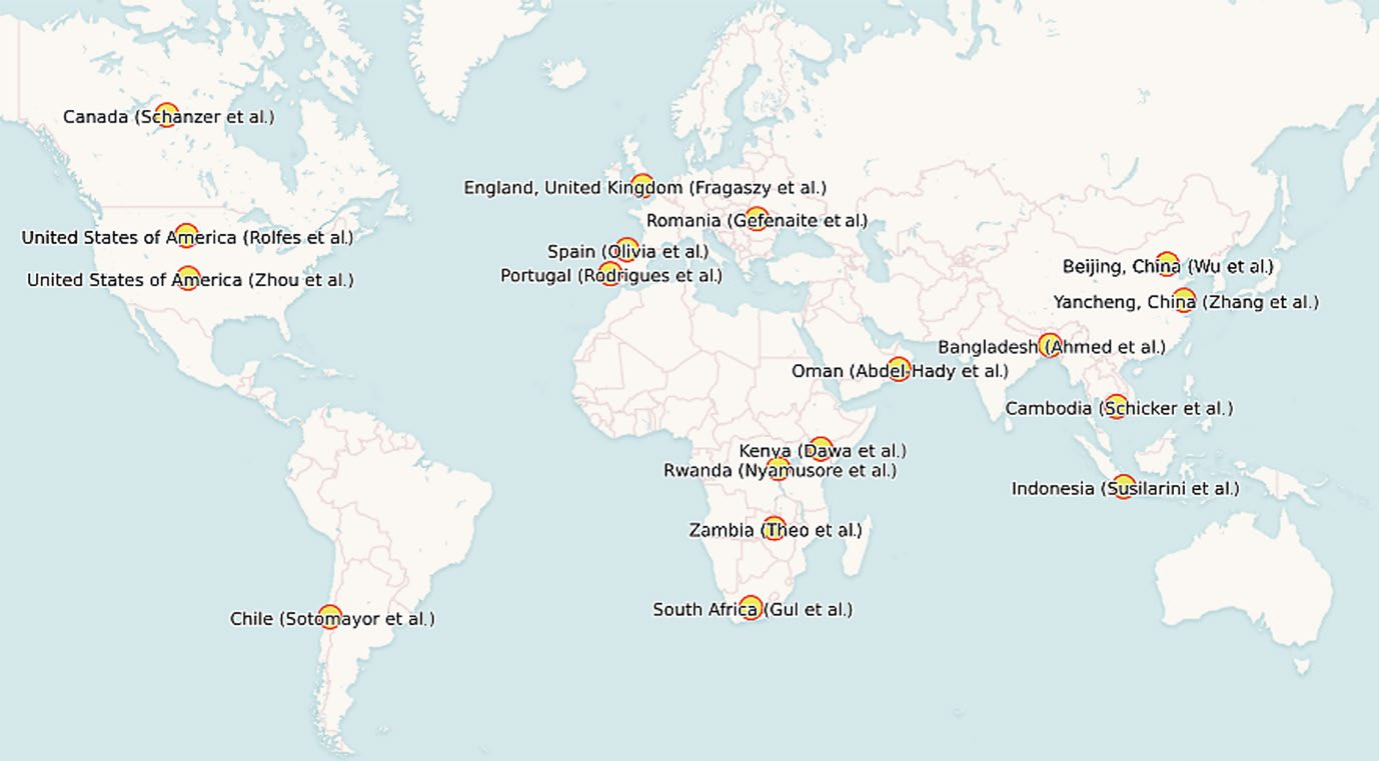
Locations of population‐specific articles in this Special Edition of the journal

Eighteen population‐specific articles have been included in this Special Edition—including five from Asia,^18^, ^19^, ^20^, ^21^, ^22^ four from Africa,^23^, ^24^, ^25^, ^26^ four from Europe,^27^, ^28^, ^29^, ^30^ three from North America,^31^, ^32^, ^33^ and one each from South America^34^ and the Middle East.^35^ (Figure 1 and Table 1) This reflects a broad geographical breadth of new research, and these data will add to our understanding of the burden of disease worldwide, especially in LMICs.

**Table 1:** Summary of country‐specific burden of disease studies in the special edition

Country	Burden of disease measures	Period	Used WHO Manual	Representativeness of entire country	Key results	Reference
Bangladesh	Influenza‐associated deaths	2010‐2012	Yes; adapted for calculation of annual mortality rates	Yes; 11 sentinel surveillance sites spanning all administrative divisions.	Among 4221 surveillance case‐patients, 553 (13%) were positive for influenza viruses. The influenza‐associated mortality rate was 6 (95% CI 3‐14) per 100 000 in 2010‐11, and 11 (95% CI 2‐25) per 100 000 in 2011‐2012. Rates of influenza‐associated deaths were highest in those aged above 60 y.	18
Cambodia	SARI	2015	Yes; HSA approach used	No; one sentinel surveillance site extrapolated to the provincial population	Adjusted influenza‐associated 2015 SARI hospitalization rate was 13.5/100 000 persons.	19
Beijing, China	Influenza‐associated excess deaths	2007‐2013	No	No; sentinel hospitals and national‐level mortality data were used.	2375 (CI 1002‐8688) deaths attributed to influenza per season. Mortality rate associated with the 2009 H1N1 pandemic in 2009/2010 was comparable to that of seasonal influenza (19.9 [CI 10.4‐33.1] vs 17.2 [CI 7.2‐67.5] per 100 000).	20
Yancheng, China	Influenza‐attributable excess resp. deaths	2011‐2015	No	No; surveillance data of sentinel sites in Yancheng taken from national system.	Annual average excess respiratory deaths of 4.59 (95% confidence interval: 3.94, 7.41) per 100 000 persons associated with influenza. Almost all occurred in persons ≥65 y.	21
Indonesia	SARI	May 2013‐April 2016	Yes; HAS to estimate sentinel hospital catchment populations	No; only sentinel hospitals in three districts.	Annual incidence of influenza‐associated SARI ranged from 13‐19 per 100 000 population. Incidence was highest in children aged 0‐4 y (82‐114 per 100 000 population), followed by children 5‐14 y (22‐36 per 100 000 population).	22
Kenya	SARI	2012‐2014	No	No; base rates from one regional hospital to extrapolate to other regions in the country.	The mean annual rate of hospitalized influenza‐associated SARI was 21 (95% CI 19‐23) per 100 000 persons, and non‐hospitalized rate was 82 (95% CI 74‐90) per 100 000 persons.	23
Rwanda	SARI hosp.	January 2012‐December 2014	Yes; used to estimate SARI hosp. rates	Yes; hosp. obtained from all public hospitals, influenza virus surveillance conducted at 6 sentinel hospitals in 5 provinces.	SARI cases accounted for 70.6% (9759/13 813) of respiratory admissions. Influenza virus detection rate was 6.3% (190/3022). Mean annual national number of influenza‐associated SARI hospitalizations was 3663 (95% CI: 2930‐4395‐rate: 34.7 per 100 000 95% CI: 25.4‐47.7).	24
South Africa	Influenza‐associated deaths	2009‐2013	No	Yes; using publicly available data on causes of death.	Annual mean mortality estimates ranged from 2.58 (95% CI 1.90‐3.25) to 4.66 (95% CI 2.03‐7.30) per 100 000 population.	25
Zambia	SARI hosp.	2011‐2014	Yes; used to estimate SARI hosp. rates	No; data from University Teaching Hospital situated in Lusaka Province extrapolated to remaining 9 provinces.	SARI cases accounted for 77.1% (13 389/17 354) of respiratory admissions. Influenza virus detection rate was 5.5% (152/2734). The mean annual national number of influenza‐associated SARI hospitalizations was 6181 (95% CI: 4321‐8041‐rate: 43.9 per 100 000; 95% CI: 30.7‐57.1)	26
Portugal	Pneumonia and influenza (P&I) excess hosp.	1998‐2015	No	Yes; data from the National Hospital Discharge database and the National Influenza Reference Laboratory.	Average excess P&I hospitalizations/season was 19.4/100 000 (range 0‐46.1/100 000), and higher excess was observed in young children less than 2 y (79.8/100 000) and ≥65 y (68.3/100 000).	27
Romania	Influenza‐associated ILI SARI hosp.	2011‐2016	Yes; used national surveillance data	Yes; national sentinel ILI surveillance conducted in all 41 counties and the capital city.	Annual incidence of ILI and influenza‐associated ILI per 100 000 persons varied between 68 (95% CI 61‐76) and 318 (95% CI 298‐338), and between 23 (95% CI 19‐29) and 189 (95% CI 149‐240), respectively. SARI incidence per 100.000 persons was 6 (95% CI 5‐7) to 9 (95% CI 8‐10), of which 2 (95% CI 1‐2) to 3 (95% CI 2‐4) were due to influenza.	28
Spain	Weekly influenza rates ILI and MCIC Hosp. rates SHCIC ICU admissions and deaths.	2010‐2016	Yes; used influenza surveillance data to estimate national burden of disease	Yes; sentinel surveillance network comprising physicians in 17 of 19 Spanish regions, and the network‐affiliated laboratories.	The highest rates of MCIC observed in <15 y (1395‐3155 cases/100 000 population in 5‐14 y) and the lowest in ≥65 y (141‐608 cases/100 000 population). SHCIC rates had a U‐shaped distribution, with annual average hospitalization rates of 16.5. Annual estimated average of 866 868 cases of ILI in primary care (55% were MCIC), 3616 SHCIC, 1232 ICU admissions and 437 deaths in SHCIC.	29
England, United Kingdom	QALD lost QALY lost Community ARI & ILI absences	2006‐2011	No	No; households recruited from 146 volunteer general practices only across England.	Average QALD lost was 0.26, 0.93, 1.61 and 1.84 for ARI, ILI, H1N1pdm09 and influenza B cases, respectively. Community influenza cases lost 24 300 QALYs in 2010/2011 and had 2.9 million absences per season from 2006/2007‐2009/2010.	30
Canada	Influenza, RSV and ORV attributable excess resp. hosp.	September 2003‐August 2014	No	Yes; hospital discharge records from Canadian Institute of Health Information Discharge Abstract Database.	33 (95% CI: 29, 38), 27 (95% CI: 22, 33) and 27 (95% CI: 18, 36) hospitalizations per 100 000 population per year attributed to influenza, RSV and ORV. Influenza virus identified in 78% (95% CI: 75%, 81%) and 17% (95% CI: 15%, 21%) of respiratory hospitalizations attributed to influenza for children and adults.	31
United States	Symptomatic community illnesses OPV Hosp. rates Deaths	2010‐2016	No	Yes; population‐based surveillance in various states, a nationwide behaviour survey, data from National Center for Health Statistics and national virological surveillance.	Influenza‐related illnesses during influenza seasons estimated to range from 9.2 million to 35.6 million, including 140 000 to 710 000 hospitalizations.	32
United States	Influenza‐associated OPV	2001‐2010	No	Yes; electronic health data from 6 of 8 integrated healthcare delivery organizations. Viral surveillance from major laboratories in 3 US regions (East, North and Central).	Outpatient rates with pneumonia visits were 39 (95% confidence interval [CI], 30‐70) and 203 (95% CI, 180‐240) per 10 000 person‐years, respectively, for interpandemic and pandemic seasons. Rates with respiratory visits were 185 (95% CI, 161‐255) and 542 (95% CI, 441‐823) per 10 000 person‐years.	33
Chile	SARI resp. hosp. and deaths due to influenza and pneumonia	January 2012‐December 2014	Yes; used SARI sentinel surveillance data	Yes; national‐level data. Records from 6 SARI sentinel sites across 3 of the 4 geographic‐administrative macro zones (covering >80% of population).	Average annual rate of hospitalizations was 71.5 (CI 95% 67.0‐76.4) per 100 000 person‐years in children < 5 y of age, 11.8 (CI 95% 11.3‐12.4) per 100 000 person‐years in people between 5 and 64 y, and 156.0 (CI 95% 150.2‐162.0) per 100 000 person‐years in adults ≥ 65 y. Annual mortality rate was 0.08 (CI 95% 0‐0.5), 0.3 (CI 95% 0.2‐0.4) and 22.8 (CI 95% 20.7‐25.2) per 100 000 person‐years for the respective age‐groups.	34
Oman	Influenza‐associated hosp. and in‐hospital deaths	January 2012‐December 2015	No	Yes; national‐level data from 11 regional hospitals.	19 405 influenza‐associated hospitalization and 847 deaths identified from 2012 to 2015. Influenza positivity percentage ranged from 6.4% in 2013 to 20.6% in 2015. Influenza‐associated hospitalization rate was 7.3 (95% CI: 6.4‐8.1) per 100 000 in 2013 and 27.5 (95% CI: 25.9‐29.1) in 2015.	35

ARI, acute respiratory infections; HAS, Hospital Admission Survey; Hosp., hospitalization; ICU, intensive care unit; ILI, influenza‐like illness; MCIC, mild confirmed influenza cases; OPV, outpatient visits; ORV, other respiratory viruses; QALD, quality‐adjusted life days; QALY, quality‐adjusted life years; Resp., respiratory; SARI, severe acute respiratory infections; SHCIC, severe hospitalizations of confirmed influenza cases.

The heterogeneity of study designs is apparent and expected. Eight studies used the WHO manual in developing their methodology,^18^, ^19^, ^22^, ^24^, ^26^, ^28^, ^29^, ^34^ although this ranged from hospital admission surveys to adapting suggested analytic methods. Most studies brought together information from various sources, including primary data from sentinel sites, provincial or national databases, and virological surveillance networks. Statistical models used range from regression analyses to semi‐parametric generalized additive models. The diversity of methods shows that there are different ways in which countries can develop burden estimates, depending on the resources available.

Two studies in this Special Edition, from India and South Africa, evaluated various data sources and methods and determined the best approaches for developing disease burden estimates in their country. The study from India found that while the Sample Registration System provided the most appropriate national mortality data set, other mortality data sources could be used for subregional estimates.^36^ The South African study showed that weekly proportion and influenza subtype‐specific proxies provided the best model fit with non‐significant differences in the estimates.^25^

The outcomes also varied across studies. The most common outcomes were deaths attributable to influenza, used in nine studies,^18^, ^20^, ^21^, ^25^, ^29^, ^32^, ^34^, ^36^ and severe acute respiratory infections (SARI) or its equivalent, used in eight studies.^19^, ^22^, ^24^, ^26^, ^28^, ^29^, ^34^ Another seven studies measured excess or absolute hospitalizations due to influenza,^27^, ^28^, ^29^, ^31^, ^32^, ^34^, ^35^ and five studies measured community or outpatient visits.^28^, ^29^, ^30^, ^32^, ^33^ Some studies were less representative for the entire country than others—there were 10 studies that collected data from across the country, although most national estimates were calculated with the help of extrapolations and appropriate adjustments for demographic differences.^18^, ^24^, ^27^, ^29^, ^31^, ^33^

Among the wealth of information in this supplement, five countries measure their influenza burden for the first time—Cambodia, Chile, Romania, Rwanda and Zambia.^19^, ^24^, ^26^, ^28^, ^34^ Fresh perspectives on influenza burden are also offered by the other studies. For example, studies from the United States and the United Kingdom shed light on the burden of disease in the community, including its effect on quality‐adjusted life days and years.^30^, ^32^, ^33^

This Special Edition also includes a review of the risk factors for severe outcomes associated with influenza illness in HICs versus LMICs, showing differences in determinants for severe outcomes between the two settings. Pregnancy, living with HIV or AIDS and young age were found to be additional risk factors in LMICs but not in HICs. Furthermore, children with neurological conditions in LMIC also had a higher risk of severe outcomes than those from HICs.^37^

Lastly, to assist countries in translating their burden of disease data into economic and policy decisions on the use of vaccines, WHO has also developed an economic evaluation tool. This is described in a paper in this Special Edition, which summarizes the key components of the tool and its implications for public health.^38^

## 4 THE WAY FORWARD

The diversity in methods used across various burden of disease studies is reflective of the multifaceted and complex nature of these estimations. Certain methods may be easier to implement in some contexts than others. For example, compared to hospitalizations, collecting accurate community‐based data on influenza may be challenging in dispersed populations or less‐resourced health systems. The different perspectives from the two studies in China also remind us that disease burden may vary within large countries with diverse populations.^20^, ^21^

Likewise, there may be various factors behind whether countries use the approach described in the WHO manual for their estimations. Some methods and measures used in countries may have pre‐dated the availability of the WHO manual or deemed to be a better fit for the country's needs. Furthermore, extrapolation and modelling may be helpful where primary data are not readily available, and there are different ways in which countries may choose to do so. The WHO manual therefore serves as a useful reference for those conducting burden of disease studies for the first time, and for others looking to improve their future estimates.

Accurate burden of disease estimates is still dependent on the quality of primary data, and this is best obtained through robust local surveillance systems. As they also support early detection and response to emerging threats, strengthening national surveillance capabilities is in the interest of global health security and in line with capability building under the International Health Regulations.^39^ In this regard, there is room for additional support to be provided to countries encountering difficulties in setting up surveillance systems and burden of disease estimates, either in the form of financial or professional expertise. The increasing number of studies in less studied regions allows for some extrapolation to neighbouring countries, but there are limitations. Extrapolation may be unavoidable for complex parameters, but each country and subregion is different and surveillance data are still required to provide a meaningful baseline.

Knowing the national and global burden of disease of seasonal influenza is an important first step in providing clarity on the magnitude of the problem at hand. However, its true utility is when such information is used to guide public health actions. There is a need for guidance to support countries to translate these data into policies and practices that would help to reduce this burden. The WHO economic evaluation tool described above is one such example.^38^ By knowing the cost‐effectiveness of different possible interventions, countries would have greater clarity on which measures should be prioritized, implemented and monitored.

Burden of disease studies are likely to continue to gain increased attention, especially with the increased emphasis on value‐based health care and cost‐effective decision‐making. Each study represents a piece to a larger puzzle, and the collective wealth of country experiences contribute to our better understanding of the overall global burden of disease of seasonal influenza, enable capacities to be built that can be used during a pandemic, and help inform us of how best to safeguard public health as individual nations and as part of a global community.
